# Impact of Body Mass Index and advanced maternal age on *in vitro* fertilization outcomes

**DOI:** 10.5935/1518-0557.20230041

**Published:** 2023

**Authors:** Hanne Lise Chaves, Mateus José Schmitz, Camila Dutra de Souza Francisquini, Vinicius Bonato da Rosa, Alessandro Schuffner, Janaina de Almeida Furlan

**Affiliations:** 1 Positivo University - Curitiba, Paraná, Brazil; 2 Conceber Clinic - Reproductive Medicine Center, Curitiba, Paraná, Brazil; 3 Brown Fertility - Florida Fertility Clinics, Jacksonville, Florida, United States; 4 Post Graduate Program in Gynecology and Obstetrics, Federal University of Paraná, Curitiba, Brazil

**Keywords:** body mass index, embryo transfer, *in vitro* fertilization, maternal age, pregnancy rate

## Abstract

**Objective:**

To evaluate the impact of body mass index associated with advanced maternal age on pregnancy outcomes.

**Methods:**

A retrospective and observational study that included 808 *in vitro* fertilization cycles and evaluated: age, weight, height, number of oocytes and mature oocytes, number of embryos and transferred embryos, fertilization and clinical pregnancy rates. Four categories of body mass index: underweight, adequate weight, overweight and obesity. We classified age into 4 categories: 35-37; 38-40; 41-42 and over 42 years of age. The means and rates were calculated and compared between different ages and body mass index groups.

**Results:**

For the fresh group, women who achieved clinical pregnancy had a lower mean age than those who did not become pregnant, being the higher the pregnancy rate the lower the age (*p*<0.0001). After logistic regression analysis for data associated with clinical pregnancy in the fresh group, the number of transferred embryos remained higher in the overweight category (*p*=0.0001). Overweight and obese women had a significantly higher rate of mature oocytes when compared with adequate weight (*p*=0.015). Analysis using the ROC curve indicated an area under the curve of 60% (*p*=0.002) for the fresh group.

**Conclusions:**

The adverse effect of high BMI on clinical pregnancy rates is greater in women under 35 years compared to older women; and age had a higher impact on live birth rate rather than BMI, when the analysis was performed on older women, with the impact of BMI on the probability of having a live birth depending on maternal age.

## INTRODUCTION

The prevalence of obesity and overweight has increased more and more in recent years. According to the Surveillance of Risk Factors and Protection for Chronic Diseases by Telephone Survey (Vigitel), carried out by the Brazilian Ministry of Health, in 2010, the percentage of women over age 18 with excess weight (Body Mass Index - BMI ≥ 25 kg/m^2^) was 44.3%, and obese (BMI ≥ 30 kg/m^2^) was 15.6%. In 2019, these percentages increased to 53.9% and 20.7%, respectively. Additionally, the study reported that overweight and obesity was directly proportional to the woman’s age (Brazil, 2020). In addition to this trend, high weight is a frequent cause of infertility in women of reproductive age (Klenov & Jungheim, 2014) in addition to increasing the gestational risk, predisposing to gestational hypertension, preeclampsia, gestational diabetes, and cesarean delivery (Norman & Reynolds, 2011; El-Chaar *et al*., 2013; Triunfo & Lanzone, 2014; Kumbak *et al*., 2012).

In addition to the difficulties related to infertility, a reduction in the rates of implantation, clinical pregnancy and live births in cycles with autologous oocytes was observed in women with high BMI undergoing in vitro fertilization (IVF), compared to women with adequate BMI (18.5-24.9kg/m^2^). In addition, the probability of miscarriage was also higher in women with a higher BMI (Kumbak *et al*., 2012).

Thus, it may seem advantageous to invest in BMI reduction before an IVF procedure; however, this may not be true in the cases of older women, as they also deal with another major factor of infertility: age. Excessive fat delays time to pregnancy, and as consequence, the increase in time leads to advanced maternal age (Best & Bhattacharya, 2015).

The decline in a woman’s reproductive function can happen very quickly as the years progress, regardless of her weight; the prevalence of aneuploidies, one of the factors responsible for this fact, increases rapidly after the age of 38 and from then onwards, a difference of two years can determine a significant reduction in the chances of a woman getting an euploid embryo (Franasiak *et al*., 2014).

Therefore, the time for a woman to achieve weight reduction may be long enough for age-related difficulties to appear (Klenov & Jungheim, 2014). To clarify this dilemma, it is necessary to determine whether overweight and obesity have such an impact on the fertility of older women, as much as they do for their younger counterparts.

As we improve our understanding of the obesity impact on women in advanced maternal age, it is possible to develop novel treatment strategies, aiming at an individualized treatment. The aim of this study was to evaluate the impact of BMI associated with advanced maternal age on IVF outcomes.

## MATERIAL AND METHODS

### Experimental Design

The present study was observational and retrospective through the review of medical records of patients undergoing *in vitro* fertilization at a private *in vitro* fertilization clinic in Curitiba, Paraná, Brazil.

### Selected Patients

Inclusion criteria were all cycles of patients diagnosed with female, male or both infertility factors, undergoing IVF using the Intracytoplasmic Sperm Injection (ICSI) technique for oocyte fertilization, women who were 35 years of age or older, semen homologous or heterologous. Cycles with incomplete medical records and cycles with oocytes of heterologous origin were used as exclusion criteria.

The diagnosis of infertility was given to women who did not become pregnant after 6 months of regular unprotected attempts, as defined by the American Society for Reproductive Medicine for women aged 35 years and older (Practice Committee ASRM, 2013).

The data came from coded spreadsheets from the co-participating institution’s database, in order to guarantee the secrecy of all information. Data were grouped into three spreadsheets according to the type of IVF cycle performed. The first worksheet, called FRESH, concentrated the fresh cycles, in which the embryonic transfer (ET) was performed in the same cycle of ovarian stimulation and follicular aspiration. The second worksheet, FET, corresponds to the cycles in which frozen embryos were transferred (Frozen Embryo Transfer). The third spreadsheet gathered the fresh cycles with ovarian stimulation and follicular aspiration, but there was no embryo transfer, and they were frozen (Freeze all).

### Study Groups

After applying the inclusion and exclusion criteria, 808 cycles were evaluated, with FRESH (n=472); FET (n=130) and Without ET (n=206).

The data for each group shown above were grouped into four BMI categories according to the Centers for Disease Control and Prevention (CDC) definition: underweight (BMI ≤ 18.5 kg/m^2^), adequate weight (BMI between 18.6 and 24.9 kg/m^2^), overweight (BMI between 25 and 29.9 kg/m^2^) and obese (BMI ≥ 30 kg/m^2^) (CDC, 2021). Also, categorization by age was adopted according to the cutoff proposed by the SART (Society for Assisted Reproductive Technology): 35 to 37 years; 38 to 40 years-old; 41 to 42 years old and over 42 years of age. The sample number of each group for the variables BMI and Age are represented in the tables of results.

### Analyzed Variables and IVF Outcomes

**Figure 1:**
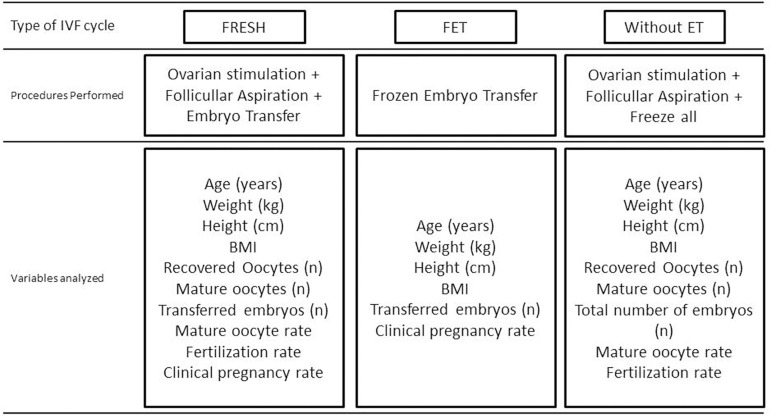
Flowchart of assessments within each experimental group.

The analyzed variables were age, weight, height, number of aspirated oocytes, number of mature oocytes (MII or metaphase II), total number of embryos, number of transferred embryos. And the IVF outcomes considered were mature oocyte rate, fertilization rate and clinical pregnancy rate. In Figure 1, we have the representation of which variables were evaluated in each group.

### Approval from the Ethics Committee

The project was submitted to the Research Ethics Committee through the Platform of the National Research Ethics Commission (CONEP), having been approved under number CAAE 34054820.5.0000.0093.

### Statistical analysis

The means and rates of each variable were calculated separately for each of the worksheets, comparing data from different age groups and different BMI groups. The statistical evaluation approach based on the type of treatment, as described, was carried out with the objective of identifying the influence of obesity in all treatment formats and at all times that the IVF patient may be submitted.

Data were submitted to statistical analysis using the Kolmogorov-Smirnov and Shapiro-Wilk tests to assess the normality of quantitative data using the GraphPad Prism 3.0 software. The Chi-square test and Fisher’s exact test were used to compare qualitative data using the SPSS 17.0 software. Continuous variables were compared using a non-parametric Mann-Whitney test for independent samples.

Binary logistic regression was used to correct the associated values, considering the covariates associated with clinical pregnancy as independent variables, STATA v.9.2 (StataCorp, USA). *p* values lower than 0.05 were considered statistically significant.

## RESULTS

The analysis of the primary outcome, clinical pregnancy rate, for the FRESH group showed that women who achieved clinical pregnancy had a lower mean age than those who did not become pregnant, with the higher the pregnancy rate the lower the age (*p*<0.0001). The mean number of transferred embryos was higher in the group that reached pregnancy compared to the group that did not (*p*<0.0001). There was no difference in the pregnancy rate between the different BMI categories (*p*=0.584). For the FET group, the clinical pregnancy rate was not influenced by age, number of transferred embryos and BMI category (*p*>0.05 for the aforementioned comparisons) (Table 1).

**Table 1:** Gestacional outcomes.

Clinical pregnancy	Age (y) (mean±SD)	No. Transferred embryos (mean±SD)	Age groups n (%)				BMI n (%)		
			35 to 37y	38 to 40y	41 to 42y	> 42y	Healthy weight	Overweight	Obese
**FRESH (n=472)** No pregnancy Clinical pregnancy ***p***	38.7±2.7 37.4±1.9 **<0.0001**	2.1±0,9 2.4±0.7 **<0.0001**	**n=204** 138(67.6) 66 (32.4)	**n=180** 140(77.8) 40 (22.2)	**n=54** 49(90.7) 5 (9.3)	**n=34** 33(97.1) 1 (2.9)	**n=291** 223 (76.6) 68 (23.4)	**n=130** 101 (77.7) 29 (22.3)	**n=51** 36 (70.6) 15 (29.4)
			**<0.0001**				0.584		
**FET (n=130)** No pregnancy Clinical pregnancy ***p***	38.8±2.7 38.9±2.9 0.347	1.46±0.67 1.7±0.7 0.054	**n=55** 44 (80.0) 11 (20.0)	**n=43** 28 (65.1) 15 (34.9)	**n=24** 15 (62.5) 9 (37.5)	**n=8** 7 (87.5) 1 (12.5)	**n=90** 65 (72.2) 25 (27.8)	**n=31** 22 (71.0) 9 (29.0)	**n=9** 7 (77.8) 2 (22.2)
			0.191				0.922		

SD: standard deviation.

In the groups that performed embryo transfer, we observed that the number of transferred embryos was higher in the overweight category compared to adequate weight in the FRESH group (*p*=0.047), and even so there was no significant difference between the two categories insofar as clinical pregnancy rate is concerned (*p*=0.900) (Table 2). After logistic regression analysis for data associated with clinical pregnancy in the FRESH group, the number of transferred embryos remained higher in the overweight category (OR 0.46; 95% CI 0.29-0.72; *p*=0.0001). With these calculations, we report that both the age and the number of transferred embryos are independent variables to predict gestational success (that is, to reach clinical pregnancy)(Table 3).

**Table 2:** Embryo transfer results by BMI categories.

Groups/Variables	Underweight	Healthy weight	Overweight	Obese	HW x UW	HW x OW	HW x O	HW x OW+O	OW x O
**FRESH (n=472)** **Clinical pregnancy n (%)** No pregnancy Clinical pregnancy **No. Transferred embryos** (mean±SD)	- -	**n=291** 223 (76.6) 68 (23.4) 2.1±0.9	**n=130** 101 (77.7) 29 (22.3) 2.3±0.8	**n=51** 36(70.6) 15(29.4) 2.1±0.9	- -	*p*=0.900 ***p*=0.047**	*p*=0.377 *p*=0.812	*p*=0.825 *p*=0.138	*p*=0.339 *p*=0.151
**FET (n=130)** **Clinical pregnancy n (%)** No pregnancy Clinical pregnancy **No. Transferred embryos** (mean±SD)	- -	**n=90** 65 (72.2) 25 (27.8) 1.53±0.67	**n=31** 22 (71.0) 9 (29.0) 1.41±0.56	**n=9** 7 (77.8) 2 (22.2) 1.78±1.1	- -	*p*=1.000 *p*=0.502	*p*=1.000 *p*=0.680	*p*=1.000 *p*=0.692	*p*=1.000 *p*=0.489

*SD: standard deviation*

*UW: Underweight; HW: Healthy weight; OW: Overweight; O: obese.*

**Table 3:** Logistic regression for data associated with clinical pregnancy.

No pregnancy x clinical pregnancy	FRESH (n=472)	OR	95%IC	p
Age groups		0.46	0.33-0.66	**<0.0001**
No transferred embryos		0.46	0.29-0.72	**0.0001**

*OR: odds ratio*.

In the group that underwent fresh cycles (FRESH and without embryo transfer) there was no statistically significant difference between the categories of BMI in age, number of aspirated oocytes and total number of generated embryos (*p*>0.05).

However, overweight and obese women had a significantly higher rate of MII oocytes when compared to women with adequate weight (*p*=0.015). Even though there was a tendency for obese women to have a higher rate of MII oocytes compared to overweight ones, although this does not have statistical significance (*p*=0.058). Despite this, the fertilization rate was significantly higher among women with adequate weight compared to those with overweight and obesity (*p*=0.027) (Table 4).

**Table 4:** Results of fresh cycles by BMI category (FRESH + group without embryo transfer).

Groups/Variables	Underweight	Healthy weight	Overweight	Obese	HW x UW	HW x OW	HW x O	HW x OW+O	OW x O
**n**	**n=8**	**n=518**	**n=206**	**n=76**					
Age (y)^*^	38.5[36-40]	39[37-41]	39[37-40]	39[36.3-40]	*p*=0.660	*p*=0.923	*p*=0.862	*p*=0.876	*p*=0.938
Aspirated oocytes (n)^*^	10[2.5-12.5]	6[3-10]	6[3-9,2]	5[3-8]	*p*=0.482	*p*=0.827	*p*=0.254	*p*=0.739	*p*=0.250
MII oocytes (n)^*^	6.5[15-10.5]	4[2-7]	4[2-7.2]	4[2-7]	*p*=0.431	*p*=0.644	*p*=0.733	*p*=0.823	*p*=0.509
**MII oocyte rate n (%)** Oocytes in MII Non-MII oocytes	50(76,9) 15(23.1)	2713(73.0) 1002(27.0)	1126(75.0) 376(25.0)	376(79.3) 98(20.7)	*p*=0.574	*p*=0.160	***p*=0.0004**	***p*=0.015**	*p*=0.058
**Fertilization rate n (%)** Fertilized oocytes Unfertilized oocytes	37(74.0) 13(26.0)	1888(67.5) 911(32.5)	722(65.5) 381(34.5)	226(59.9) 151(40.1)	*p*=0.407	*p*=0.248	***p*=0.0054**	***p*=0.027**	*p*=0.062
Total embryos (n)^*^	4[1.5-5.7]	3[1-4]	3[1-5]	2[1 -4]	*p*=0.283	*p*=0.474	*p*=0.407	*p*=0.833	*p*=0.204

* median [interquartile range]

*UW: Underweight; HW: Healthy weight; OW: Overweight; O: Obese*.

**Figure 2:**
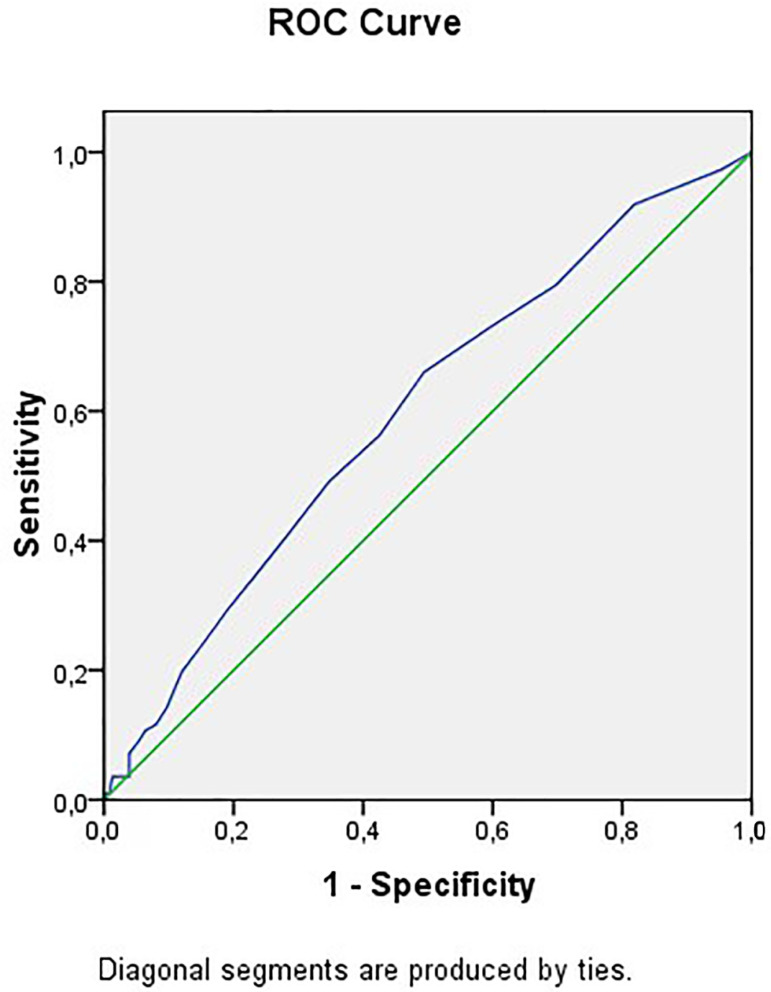
ROC curve for number of aspirated oocytes and clinical pregnancy in the FRESH group.

Analysis using the ROC curve indicated an area under the curve of 60% (*p*=0.002) for the group that underwent cycles with fresh embryo transfer (FRESH) (Table 5). The best sensitivity (66.1%) and specificity (51%) results happened using a cutoff of 5.5 or more aspirated oocytes. (Figure 2). However, there was no significant difference between the BMI categories in the chance of obtaining 5.5 oocytes or more; however, there was a strong tendency for overweight patients to have greater chances of obtaining such oocytes compared to obese patients (*p*=0.058).

**Table 5:** ROC curve cutoff analysis by BMI category.

No. Aspirated oocytes (cutoff ROC curve)	Healthy weight	Overweight	Obese	HW x OW	HW x O	HW x OW+O	OW x O
**FRESH (n=472)**	**n=291**	**n=130**	**n=51**	*p*=0.099	*p*=0.438	*p*=0.378	*p*=0.058
< 5.5	112 (38.5)	39 (30.0)	23 (45.1)				
≥ 5.5	179 (61.5)	91 (70.0)	28 (54.9)				

## DISCUSSION

This study sought to find the relationship between BMI in women of different age groups who underwent IVF. We observed that age was an important factor in reducing the clinical pregnancy rate in the FRESH group. However, the analysis with frozen embryos (FET group) showed no difference in the pregnancy rate between the age groups.

Regarding the number of aspirated oocytes, there was no difference between the BMI categories; however, the obese ones had significantly more mature oocytes compared to those with adequate weight. Despite this, the higher rate of MII oocytes obtained by obese women did not result in better fertilization rates compared to women with adequate weight. Similar results were found by the retrospective cohort which analyzed data from 1721 women in their first IVF cycle using autologous oocytes with fresh embryo transfer (Shah *et al*., 2011). This study observed that the mean number of MII oocytes was higher in the overweight and obese grade I group (BMI between 30.0 and 34.9 kg/m^2^) compared to women of adequate weight (13.5 and 13 *vs*. 12.7). Women with adequate weight had a higher mean of MII oocytes than obese ones (13.6 *vs*. 11.3); but obese women had a higher percentage of MII oocytes that failed fertilization (26.6% *vs*. 23.7%). According to the authors, the abnormalities found can either interfere with the incorporation of sperm into the oocyte or be a result of failure of the oocyte cytoplasm to carry out the condensation of chromosomes. As in the study, the penetration and incorporation of sperm was similar between the two categories of BMI, it is suggested that the fertilization failure occurred due to defects in the oocyte cytoplasm of obese women in carrying out the condensation of chromosomes (Machtinger *et al*., 2012).

However some studies demonstrate that there are no significant differences in the number of aspirated and fertilized oocytes, as well as in the number of transferred embryos between the BMI categories when using autologous oocytes, whether fresh or frozen (Legge *et al*., 2014; Sarais *et al*., 2016; Ozgur *et al*., 2019).

In our study, it did not seem to influence the clinical pregnancy rate in women aged 35 years and over, either for freshly transferred or frozen embryos. In the aforementioned study the clinical pregnancy rate was lower the higher the degree of obesity presented by patients compared to those of adequate weight (OR 0.67; 95% CI 0.46-0.97 for patients with grade I obesity and OR 0.50; 95% CI 0.31-0.82 for patients with grade III) (Shah *et al*., 2011). However, it is noteworthy that this study was carried out with younger women when compared to our sample, which was carried out exclusively with women of advanced age.

To determine the combined impact of BMI and maternal age on live birth rate, a study published recently evaluated data from 51,959 cycles with fresh autologous oocytes. The authors reported that within each age group the live birth rate declined for all BMI categories above adequate (linear trend with *p*<0.001 for all). However, this trend was less pronounced in older women, with little difference between the BMI categories when women over 38 years were analyzed. The study suggests that the impact of BMI is more important in younger ages than in older ones, and in the latter, the live birth rate is more influenced by the prevalence of aneuploidies than by BMI (Goldman *et al*., 2019).

Furthermore, both the BMI and the FET protocol used were shown to be significant predictors of success in the treatment of obese women. For example, the inadequate effect of endogenous progesterone may help explain the reason for the high rate of miscarriages found in the overweight group, whose luteal phase support protocol was performed with progesterone via the vaginal route alone (Tremellen *et al*., 2017).

A US meta-analysis investigated the results of the use of donated oocytes in obese recipients and concluded that there is no difference between obese and non-obese women in pregnancy, implantation, abortion and live birth rates when using donated oocytes. However, it is interesting to note that the recipients in this study received luteal phase support with high doses of intramuscular progesterone, which could potentially mask any deficits (Jungheim *et al*., 2013). On the other hand, another study which assessed the impact of using donated oocytes in obese women, reported a significant reduction in implantation, pregnancy and live birth rates as the woman’s degree of obesity increased (Bellver *et al*., 2013).

A study which compared IVF results among women over and under 35 years of age reported that for women under 35 years, there was no difference between the BMI categories in the number of MII oocytes (*p*=0.087) and embryos (*p*=0.099), fertilization rates (*p*=0.101) and clinical pregnancy (*p*=0.311). In the group of women older than 35 years, the clinical pregnancy rate was lower in the obese group compared to their adequate weight counterparts (10% *vs*. 24.5%; *p*=0.02), but the number of M-II oocytes (*p*=0.959), embryos (*p*=0.984) and fertilization rate (*p*=0.945) did not differ between the BMI categories. Thus, the authors suggest that different stages of IVF can be affected depending on the age of the woman seeking treatment (Vural *et al*., 2016).

The rate of MII oocytes was lower in obese patients compared to those of adequate weight (*p*=0.005), but the pregnancy rate did not differ between the BMI categories even after adjusting for maternal age (Sarais *et al*., 2016). However, the rate of abortions in overweight patients was higher than in those of adequate weight (OR 2.5; 95% CI 1.02 to 6.14; *p*=0.04). Thus, the lower number of MII did not impact the number and quality of transferred embryos, as well as the clinical pregnancy rate between the different BMI groups. The authors suggest that the quality and number of embryos does not seem to be affected by BMI, but the number of abortions does.

A prospective randomized controlled trial conducted a weight reduction program in infertile women aged 18 to 38 years with a BMI between 30 and 35 prior to IVF treatment to determine whether weight reduction increases live birth rates in this population. The fertilization rate did not differ between the groups (*p*=0.40) and neither did the number of transferred embryos (*p*=0.22). However, some factors may have confounded the results, such as the fact that the intervention group took longer to perform IVF due to the time invested in weight reduction.

In our study, the number of transferred embryos was significantly higher for the overweight category in relation to those with adequate weight, but that did not result in a higher pregnancy rate for overweight women, suggesting that the mechanisms by which overweight leads to subfertility vary far beyond embryos in terms of quality and number.

A retrospective study, that included 1528 cycles of in vitro fertilization with autologous oocytes, analyzed morphological and morphokinetic aspects in the development of 1366 embryos from women classified in different BMI categories. After following the standard protocols for culture and embryo transfer, the study found no difference between the BMI categories in embryo morphology, assessed according to embryo quality scores, and in pregnancy rates (*p*>0.05 for the two variables). However, embryos from obese women had more than one time delay in cleavage compared to those from adequate weight women (*p*<0.01), suggesting that there is slower embryo development associated with maternal overweight and obesity. The authors reiterate the fact that they selected for transfer the embryos that developed faster in both categories of BMI (adequate and obese), and this may have minimized differences in embryo quality and, consequently, in pregnancy rates (Bartolacci *et al*., 2019).

The study by Shah *et al*. (2011), previously mentioned, also performed a morphological analysis of the embryo and found no differences between the BMI categories, but this did not prevent the pregnancy rate from being lower in obese women. The authors suggest that being overweight can have a deleterious effect on the reproductive process regardless of embryonic quality, or that the way in which obesity affects embryonic quality may not be related to morphological characteristics (Shah *et al*., 2011).

Our study may be limited by its retrospective nature, which does not allow control of pre-recorded information, not allowing to detect changes in BMI during treatment, since anthropometric data were measured only once. Another of the main limitations is the relatively small sample size of the high BMI group when only advanced age was included. However, the presence of detailed clinical information allowed us to partially compensate for some of these limitations; this study actually represents one of the few conducted in a population with exclusively advanced maternal age. A strong point is also the fact that we have separated the sample between fresh transfers and frozen embryos, minimizing possible biases.

Another potential limitation is the fact that a less specific marker of body composition, such as BMI, was used, since it does not distinguish the proportion between lean and fat mass in weight composition. One measure that could be used to reduce this bias is waist circumference, which is highly related to fat mass and central obesity.

Based on our results, the differences were not significant due to the greater influence of age in relation to weight for some of the variables. In general, we could observe that the adverse effect of high BMI on clinical pregnancy rates is greater in women under 35 years compared to older women. In addition, age has a higher impact on live birth rate rather than BMI when the analysis is performed on older women, with the impact of BMI on the probability of having a live birth depending on maternal age.
